# Biophysical Characterization of Nucleophosmin Interactions with Human Immunodeficiency Virus Rev and Herpes Simplex Virus US11

**DOI:** 10.1371/journal.pone.0143634

**Published:** 2015-12-01

**Authors:** Kazem Nouri, Jens M. Moll, Lech-Gustav Milroy, Anika Hain, Radovan Dvorsky, Ehsan Amin, Michael Lenders, Luitgard Nagel-Steger, Sebastian Howe, Sander H. J. Smits, Hartmut Hengel, Lutz Schmitt, Carsten Münk, Luc Brunsveld, Mohammad R. Ahmadian

**Affiliations:** 1 Institute of Biochemistry and Molecular Biology II, Medical Faculty, Heinrich-Heine University, Düsseldorf, Germany; 2 Laboratory of Chemical Biology & Institute of Complex Molecular Systems, Department of Biomedical Engineering, Technische Universiteit Eindhoven, Eindhoven, Netherlands; 3 Clinic for Gastroenterology, Hepatology and Infectiology, Medical Faculty, Heinrich-Heine University, Düsseldorf, Germany; 4 Institute of Biochemistry, Heinrich-Heine University, Düsseldorf, Germany; 5 Institute of Complex Systems (ICS-6), Research Centre Jülich, Jülich, Germany; 6 Institute of Physical Biology, Heinrich-Heine University, Düsseldorf, Germany; 7 Institute of Virology, Medical Faculty, Heinrich-Heine University, Düsseldorf, Germany; 8 Institute of Virology, University Medical Center Freiburg, Freiburg, Germany; University of Regensburg, GERMANY

## Abstract

Nucleophosmin (NPM1, also known as B23, numatrin or NO38) is a pentameric RNA-binding protein with RNA and protein chaperon functions. NPM1 has increasingly emerged as a potential cellular factor that directly associates with viral proteins; however, the significance of these interactions in each case is still not clear. In this study, we have investigated the physical interaction of NPM1 with both human immunodeficiency virus type 1 (HIV-1) Rev and Herpes Simplex virus type 1 (HSV-1) US11, two functionally homologous proteins. Both viral proteins show, in mechanistically different modes, high affinity for a binding site on the N-terminal oligomerization domain of NPM1. Rev, additionally, exhibits low-affinity for the central histone-binding domain of NPM1. We also showed that the proapoptotic cyclic peptide CIGB-300 specifically binds to NPM1 oligomerization domain and blocks its association with Rev and US11. Moreover, HIV-1 virus production was significantly reduced in the cells treated with CIGB-300. Results of this study suggest that targeting NPM1 may represent a useful approach for antiviral intervention.

## Introduction

**Fig 1:**
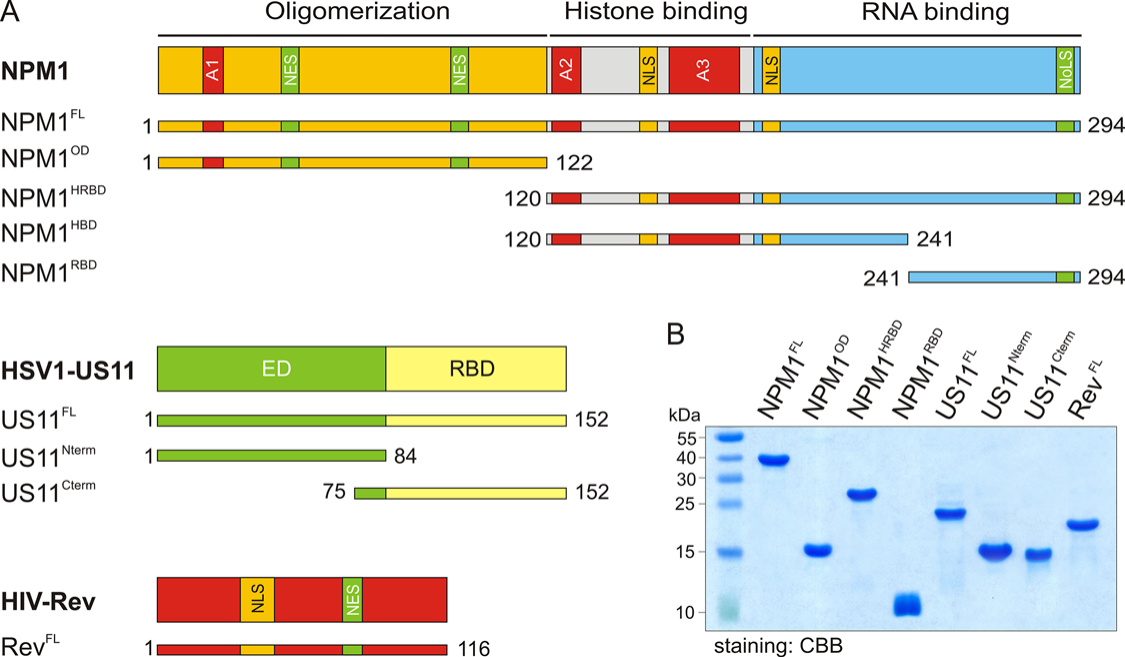
Schematic representation of domain organization, various constructs and proteins of NPM1, HSV-1 US11, and HIV-1 Rev. (A) Domains and various constructs of NPM1, US11 and Rev. The numbers indicate the N- and C-terminal amino acids of the respective constructs used in this study. A1-A3, acidic regions 1–3; Cterm, C-terminal; ED, effector domain; FL, full-length; HRBD, histone and RNA-binding domains; HBD, histone binding domain; NES, nuclear export signal; NLS, nuclear localization signal; NoLS, nucleolar localization signal; Nterm, N-terminal; OD, oligomerization domain; RBD, RNA-binding domain. (B) Coomassie brilliant blue (CBB) stained SDS-PAGE of purified proteins used in this study.

Nucleophosmin (NPM1, also known as B23, numatrin, NO38) is a multifunctional phosphoprotein, predominantly localized in the nucleoli, which participates extensively in RNA regulatory mechanisms including transcription, ribosome assembly and biogenesis, mRNA stability, translation and microRNA processing [1, 2]. NPM1 (294 amino acids; 37 kDa) consists of an N-terminal oligomerization domain (OD), a central histone binding domain (HBD) and a C-terminal RNA-binding domain (RBD) (Fig 1A) [3]. It also contains nuclear localization signals (NLSs) at the N-terminus, central nuclear exports signals (NESs) and a nucleolar localization signal (NoLS) at the very C-terminus (Fig 1A). NPM1 shuttles between the nucleus and cytoplasm and accordingly, a proportion of nucleolar NPM1 constantly translocates to the nucleoplasm and inner nuclear membrane as well as to the cytoplasm and inner and outer plasma membrane [2, 4, 5]. Due to this ability, NPM1 has been implicated in many stages of viral infection through interaction with a multitude of proteins from heterologous viruses (Table 1), including Human immunodeficiency virus type 1 (HIV-1) Rev [4], Human T-cell leukemia virus type 1 (HTLV-1) Rex [6] and Herpes simplex virus type 1 (HSV-1) UL24 [7].

**Table 1:** Nucleophosmin involvement in multiple viral infections.

Virus^a^	Partner	Domain	Effect/observation	References
AAV	Rep	n.d.	Viral assembly	[58]
Adenovirus	Core protein V	n.d.	NPM1 re-localization, Replication, Viral assembly	[59, 60]
Adenovirus	Basic core protein	n.d.	Transcription, Replication	[61]
Adenovirus	Core protein V, pre-VII	n.d.	Replication, chromatin assembly	[62, 63]
CHIKV	n.d.	n.d.	n.d.	[64]
EBV	EBNA1	HBD	Transcription	[65, 66]
EBV	EBNA2	OD	Transcription, latency	[67]
EBV	EBNA3	n.d.	Transcription	[68]
EMCV	3BCD	n.d.	Nuclear transport	[69]
HBV	core protein 149	n.d.	Capsid assembly	[70–72]
HBV	X protein	n.d.	n.d.	[73, 74]
HCV	Core protein	n.d.	Transcription	[75]
HDV	Antigen	n.d.	n.d.	[76]
HIV-1	Rev	OD, HBD	n.d.	[4]; this study
HIV-1	Tat	n.d.	NPM1 acetylation, transcription	[77–79],
HRSV	Matrix protein	n.d.	Replication	[80]
HSV-1	UL24	n.d.	NPM1 re-localization	[7]
	US11	OD	n.d.	this study
HTLV-1	Rex	HBD	n.d.	[6]
JEV	Core protein	OD	Replication	[81]
KSHV	LANA	n.d.	NPM1 phosphorylation (T199), latency	[82]
NDV	Matrix protein M	RBD	NPM1 re-localization, Replication	[83]
PEDV	N protein	n.d.	Nucleolar co-localization	[84]

^a^ Virus abbreviation: AAS, Adeo-associated virus; EBV, Epstein Barr virus; CHIKV, Chikungunya virus; EMCV, Encephalomyocarditis virus; HBV, Hepatitis B virus; HCV, Hepatitis C virus; HDV, Hepatitis delta virus; HIV-1, Human immunodeficiency virus type 1; HRSV, Human respiratory syncytial virus; HSV-1, Herpes simplex virus type 1; HTLV1, Human T-cell leukemia virus type 1; JEV, Japanese encephalitis virus; KSHV, Kaposi's sarcoma-associated herpes virus; NDV, Newcastle disease virus; PEDV, porcine epidemic diarrhea virus. n.d., not determined.

Rev is 116 amino acid long and its RNA-binding domain is composed of an arginine-rich motif (ARM), which binds to various HIV-1 RNA stem loop structures [8]. The RNA- binding domain of Rev also acts as a nuclear/nucleolar targeting signal, which can deliver cytoplasmic proteins to the nucleus or nucleolus [8, 9]. Many host proteins including DDX1, DDX3, eIF5A, exportin-1, hRIP/Rab, Matrin-3, NPM1, PIMT, and RNA helicase A have been suggested to bind to Rev prior to induction of its nuclear translocation [10–13]. NPM1 interaction with Rev appears to be necessary for nucleolar localization of Rev [4]. In fact, the HIV-1 Rev response element, a segment of viral RNA, represents a nuclear export signal, which triggers, *via* Rev binding, the nucleocytoplasmic shuttling of viral transcripts in infected cells [14]. A similar mechanism is controlled by Rex responsive element [15]. Most interestingly, US11, a protein of HSV-1, has the potential of directly binding to the Rev and Rex response elements and functionally substituting for Rev and Rex functions [4, 14].

HSV-1 virions have four morphologically separate structures, a DNA core, capsid, tegument, and envelope. Tegument proteins fill the space between the capsid and the envelope [16]. US11 is a tegument protein and approximately 600 to 1,000 molecules per virion are released in the target cell upon virus entry [17]. It is a multifunctional protein involved in posttranscriptional regulation of gene expression and in biological processes related to the survival of cells following environmental stress [18, 19]. US11 is localized in the nucleus and the cytoplasm, but especially accumulates in the nucleolus [20, 21]. It has been reported that US11 has RNA-binding activity and can associate strongly with ribosomes and has also been found in rRNA and polysome containing fractions [17, 22]. US11 also interacts with several host proteins, including nucleolin [23], ubiquitous kinesin heavy chain (uKHC) [24], homeodomain-interacting protein kinases 2 (HIPK2) [19], and protein kinase R (PKR) [25], which in turn counteracts the antiviral host defense system. Furthermore, although US11 protein is not essential for viral growth in cell cultures, it plays a vital role in the cells subjected to thermal stress [26], recovery of protein synthesis and survival in heat shock-treated cells [27].

In this study we investigated Rev-NPM1 interaction and found that Rev shows high-affinity binding to two domains of NPM1, OD and HBD, in an RNA-independent manner. Due to the functional homology of US11 with both HIV-1 Rev and HTLV-1 Rex, it was tempting to examine US11 binding to NPM1. The achievements in this study demonstrates, for the first time, a physical interaction between the C-terminal domain of US11 and NPM1^OD^ in an RNA-independent manner. The Rev and US11 association with NPM1 was prevented by a cyclic peptide, CIGB-300, which also bound to NPM1^OD^ but not to the other NPM1 domains. Cell-based experiments revealed a significant reduction of HIV-1 virus production in the presence of CIGB-300. Thus, the association of nucleolar protein NPM1 with the viral proteins Rev and US11 may advance our understanding of HIV and HSV pathology and further implies that NPM1 can be exploited as a therapeutic target for infectious diseases.

## Materials and Methods

### Constructs

The coding sequence of NPM1 full-length (NPM1^FL^, aa 1–294), kindly provided by F. Carrier [28]. Oligomerization domain (NPM1^OD^, aa 1–122), histone and RNA-binding domains (NPM1^HRBD^, aa120-294), histone binding domain (NPM1^HBD^, aa 120–241), RNA-binding domain (NPM1^RBD^, aa 241–294), HSV-1 US11 full-length (US11^FL^, aa 1–152), Nterm (US11^Nterm^, aa 1–84) and Cterm (US11^Cterm^, aa 79–152) as well as HIV-1 Rev full-length (Rev^FL^, aa 1–116) were amplified by PCR and cloned into pGEX-4T1-Ntev or pET-23b to obtain GST-fusion or His-tagged proteins. The Myc-tagged HSV-1 US11^FL^ was cloned into pcDNA3.1-Myc for expression in eukaryotic cells. pNL4-3 was used to produce replication competent HIV-1 [29].

### Cell culture

COS-7 and HeLa cells were obtained from German Collection of Microorganisms and Cell Cultures (Braunschweig, Germany). TZM-bl Cells were from NIH AIDS reagent program and HOS.CD4.CXCR4 cells were from CFAR (Centers for AIDS Research). All cells were grown in DMEM supplemented with 10% fetal bovine serum (FBS) (Life Technologies) and penicillin/streptomycin (Life Technologies) as antibiotics. Cells were grown in a humidified CO_2_ (5%) atmosphere at 37°C. Trypsin/EDTA was from Genaxxon Bioscience GmbH (Ulm, Germany).

### Antibodies and fluorescent probes

Mouse monoclonal anti-NPM1 (ab10530) recognizing the C-terminal 68-amino acids and rabbit monoclonal anti-NPM1 (ab52644) recognizing the N-terminal 122-amino acids were from Abcam (Cambridge, United Kingdom), Rabbit monoclonal anti-myc from Cell Signaling Technology, Inc. (Boston, USA), Alexa fluor 488 mouse anti-rabbit IgG and Alexa fluor 633, and goat anti-mouse IgG from Molecular Probes (Oregon, USA), and normal monoclonal Rabbit IgG (sc-2027) was from Santa Cruz Biotechnology, Texas, USA.

### Proteins

For protein expression the *Escherichia coli* strains BL21(DE3), pLysS BL21(DE3), CodonPlusRIL, or BL21(Rosetta), were transformed and used to purify the respective protein as previously described [30, 31]. All purified proteins were analyzed by SDS-PAGE (Fig 1B) and stored as either tag-fused or cleaved protein at -80°C.

### Transient transfection

COS-7 and HeLa cells were transfected using the TurboFect transfection reagent according to the manufacturer's instructions (Thermo Scientific) in 24-well plates or 10 cm dishes by using 0.5 μg or 5 μg plasmid DNA per transfection, respectively.

### Confocal laser scanning microscopy

Confocal imaging was performed using a LSM510-Meta confocal microscope (Zeiss, Jena, Germany) as previously reported [5].

### Immunoblotting

Proteins were heated in Laemmli sample buffer and subjected to SDS-PAGE. The proteins were transferred to nitrocellulose membranes (Hybond C, GE Healthcare) using Mini Trans-Blot cell (100 volt for 1 h) (BIO-RAD, USA), and immunoblotted using monoclonal primary antibody to mouse NPM1 antibody (Abcam), rabbit NPM1 antibody (Abcam), and rabbit myc antibody (Cell Signaling) for 1 h. After three washing steps, membranes were incubated with polyclonal horseradish peroxidase-coupled secondary antibodies for 1 h and signals were visualized by the ECL detection system (GE Healthcare) and images were collected using the ChemoCam Imager ECL (INTAS science imaging, Germany).

### Immunoprecipitation

COS-7 cells were transiently transfected with cDNA encoding Myc-tagged US11. After 48 h, an equal number of the cells were lysed in a buffer, containing 30 mM Tris/HCl, pH 7.5, 150 mM NaCl, 1 mM EDTA, 1% Triton X-100, 2.5 mM Na-pyrophosphate, 1 mM β-glycerophosphate, 1 mM sodium vanadate, and one EDTA-free protease inhibitor cocktail tablet (Roche, Mannheim, Germany). Lysates were centrifuged at 12,000×*g* for 2 min. The supernatant was precleared with protein G agarose (Roche, Mannheim, Germany) and divided to three parts for IgG control, beads control and IP, and then incubated with an anti-myc antibody (Cell Signaling) overnight at 4°C. Afterwards, protein G-Agarose beads were added to the lysate for 1 h before recovering the beads by centrifugation at 500×*g* for 5 min at 4°C. The beads were washed 4-times in the lysis buffer, and resuspended in Laemmli sample buffer. Precipitates and total cell lysate were subjected to SDS-PAGE, and Western blotting as described above.

### Analytical size exclusion chromatography (aSEC)

The complex formation of NPM1^OD^ and US11^FL^ was analyzed using a superdex 200 10/30 column (GE Healthcare, Uppsala, Sweden) and a buffer, containing 30 mM Tris-HCL (pH 7.5), 150 mM NaCl, 5 mM MgCl_2_, and 3 mM dithiothreitol. The flow rate was sustained at 0.5 ml/min. Fractions were collected at a volume of 0.5 ml and then peak fractions were visualized by 12.5% SDS-PAGE gel and staining using coomassie brilliant blue (CBB).

### Pull-down assay

GST, GST-fused NPM1 and HSV-1 US11 variants as well as HIV-1 Rev were expressed in *E*. *coli* and purified using standard protocols [30, 31]. In order to obtain prey proteins the GST-tag was cleaved off with purified tobacco etch virus (tev) protease and removed by reverse GSH affinity purification. Pull-down experiments were performed by adding 50 μg purified proteins, e.g. HIV-1 Rev and HSV US11 variants, or COS-7 cell lysate transfected with pcDNA-mycUS11^FL^ to 25 μg of GST-fused NPM1 proteins, immobilized on 100 μl glutathione-conjugated Sepharose 4B beads (Macherey-Nagel, Duren, Germany). The mixture was incubated at 4°C for 1 h in a buffer containing 30 mM Tris/HCl, pH 7.5, 150 mM NaCl, 5 mM MgCl_2_, and 3 mM Dithiothreitol. In cases of RNase treatments, 70 U RNase A (Qiagen, Hilden, Germany) were added to the same buffer in order to determine an RNA dependent interaction between the NPM1 variants and HIV-1 Rev. After four washing steps with the same buffer, proteins retained on the beads were heat-denatured (7 min at 90°C) and analyzed by SDS-PAGE followed by coomassie brilliant blue (CBB) staining or by Western blotting. Mixed samples prior to pull-down (PD) analysis were used as input controls.

### Isothermal titration calorimetry (ITC)

All proteins were prepared in ITC buffer, containing 30 mM Tris-HCl, pH 7.5, 150 mM NaCl, 5 mM MgCl_2_, and 1 mM Tris (2-carboxyethyl) phosphine (TCEP) on a size exclusion chromatography (SEC) column (Superdex 200, 16/60, GE Healthcare, Uppsala, Sweden). ITC measurements were performed at 25°C using a VP-ITC system (Microcal, Northampton, MA, USA) as previously reported [32]. The final data analysis was carried out using Origin software (Microcal). The experimental data were evaluated using Origin 7.0 software (Microcal) to determine the binding parameters including association constant (K_a_), number of binding sites (n), and enthalpy (ΔH). Control measurements were carried out by titrating buffer to the protein.

### Analytical ultracentrifugation (AUC)

Sedimentation velocity centrifugation experiments at 50,000 rpm and 20°C were carried out in a Beckman Optima XL-A (Beckman-Coulter, Brea, CA, USA), equipped with absorption optics, and a four-hole rotor. Samples (volume 400 μL) were filled into standard aluminum double sector cells with quartz glass windows. Measurements were performed in absorbance mode at detection wavelengths 230 nm. Radial scans were recorded with 30 μm radial resolution at ~1.5 min intervals. The software package SEDFIT v 14.1 (www.analyticalultracentrifugation.com) was used for data evaluation. After editing time-invariant, noise was calculated and subtracted. In SEDFIT continuous sedimentation coefficient distributions c(s) were determined with 0.05 S resolution and F-ratio = 0.95. Suitable s-value ranges between 0 and 20 S and f/f_0_ between 1 and 4 were chosen. Buffer density and viscosity had been calculated with SEDNTERP v 20111201 beta (bitcwiki.sr.unh.edu) [33]. The partial specific volume of NPM1^OD^ fragment, NPM1^FL^and US11^FL^ were calculated according to the method of Cohn and Edsall [34] as implemented in SEDNTERP. NPM1^OD^ was analyzed at 0.25 concentrations in 30 mM Tris-HCl, pH 7.5, 150 mM NaCl, and TCEP (1 mM). After equilibrium was reached, concentration profiles were recorded with 10 μm radial resolution and averaging of seven single registrations per radial value. Equilibria had been established at 14,000, 16,000, 25,000, 42,000 and 50,000 rpm. Data evaluation was performed using SEDPHAT.

### Multi angle light scattering (MALS)

MALS experiments were performed as described [35]. Briefly, light scattering measurement of purified NPM1^OD^ alone or combined with US11^FL^ was performed on a MALS instrument (miniDAWN™ TREOS). For exact protein mass calculation, UV absorptions at 280 nm (Agilent Infinity 1260) and refractive index (RI) signals (OptilabRex, Wyatt Technology) were collected. Raw data was analyzed and processed using ASTRA software (Wyatt Technology) to calculate molecular mass averages and polydispersity indexes of analyzed protein samples.

### CIGB-300 synthesis

The CIGB-300 peptide was synthesized at room temperature by manual solid-phase peptide synthesis using a Rink Amide resin (0.59 mmol/g loading). Briefly, the resin (200 μmole scale) was pre-swollen by suspending in 3 mL of NMP for 10 min and the N-terminal Fmoc-protecting group cleaved by treating the resin with 3 mL of a stock solution of 20% piperidine (v/v) in *N-*methyl-2-pyrrolidone (NMP) (2 x 5 min). Each amino acid coupling was performed by pre-mixing 2 mL of a 0.4 M stock solution of *O*-Benzotriazole-*N*,*N*,*N'*,*N'*-tetramethyluronium-hexafluoro-phosphate (HBTU) in NMP with 4 mL of a 0.2 M stock solution of the amino acid building block in NMP, followed by 2 mL of a 1.6 M stock solution of *N*,*N*-diisopropylethylamine (DIPEA) stock solution, also in NMP. The reaction mixture was added immediately to the resin and the reaction vessel agitated at ambient temperature for 30 min. Each amino acid coupling was performed twice. For the coupling of the fluorescein isothiocyanate (FITC) dye, an amino acid linker (Fmoc-O1Pen-OH, Iris Biotech GmbH) was first coupled to the N-terminus, the Fmoc group deprotected under standard conditions, and then the resin was incubated with 7 eq. of FITC and 14 eq. of DIPEA in DMSO at RT for 18 h. The linear peptides (with and without FITC dye) were simultaneously deprotected and cleaved from the Rink Amide resin using a 92.5/2.5/2.5/2.5 (v/v) mixture of trifluoroacetic acid (TFA)/H_2_O/triisopropylsilane (TIS)/ ethanedithiol (EDT), and then precipitated in ice-cold diethyl ether. Finally, disulfide formation was performed by stirring the crude peptide in phosphate buffer (pH 7.5) with 1% v/v DMSO at RT for 48 h to afford either CIGB-300 or fluoresceinated CIGB-300 after purification by reverse-phase HPLC using an Alltima HP C18 column (5 μm, length 125 mm, ID: 20 mm) and 0.1% trifluoroacetic acid (TFA) in H_2_O/MeCN as mobile phase. The pure peptides were analyzed by LC-MS using a Shimadzu LC Controller V2.0, LCQ Deca XP Mass Spectrometer V2.0, Alltima C18-column 125 x 2.0 mm, Surveyor AS and PDA with solvent eluent conditions: CH_3_CN/H_2_O/1% TFA. The Rink Amide resin and all amino acid building blocks were purchased from Novabiochem®. HBTU, DIPEA, NMP, HPLC-grade CH_3_CN and HPLC-grade TFA were all purchased from Biosolve B.V. Diethyl ether was purchased from Actu-All Chemicals. FITC, ethanedithiol, and triisopropylsilane were all purchased from Sigma-Aldrich. H_2_O refers to Millipore-grade distilled water. Summary of LC-MS data (ESI): CIGB-300; [M+5TFA+3H]^3+^: 1210.25 (theoretical), 1210.13 (found); [M+6TFA+3H]^3+^: 1248,26 (theoretical), 1248.20 (found); fluoresceinated CIGB-300; [M+5TFA+3H]^3+^: 1335.61 (theoretical), 1335.73 (found); [M+6TFA+3H]^3+^: 1373.95 (theoretical), 1373,60 (found).

### Fluorescence polarization

Fluoresceinated CIGB-300 (also referred to as FITC-labelled CIGB-300) was synthesized as described above. Increasing amounts of different variants of NPM1, GST-Rev, GST-US11 and GST as a negative control were titrated into FITC-labeled CIGB-300 (0.1 μM) in a buffer containing 30 mM Tris/HCl (pH 7.5), 150 mM NaCl, 5 mM MgCl_2_, 1 mM tris-(2-carboxyethyl) phosphine and a total volume of 200 μl at 25°C using a Fluoromax 4 fluorimeter. Displacement assay was performed by titrating increasing amount of Rev and US11 to the complex of NPM1 and FITC-labelled CIGB-300. The concentration dependent binding curve was fitted using a quadratic ligand binding equation.

### Virus production assay

HOS.CD4.CXCR4 were seeded in a 24 well plate with 2.5x10^4^ cells per well. One part was treated with 100 μM CIGB-300 peptide for 30 min at 37°C and one part was left untreated. Cells were infected with HIV-1 NL4-3 (MOI 1) and after 6 h cells were washed to remove input virus. Cell culture supernatant was collected 48 h and 72 h after infection. Virus titer in the supernatant was determined by infection of TZM-bl cells and luciferase measurement three days later using the *Steady-Glo Luciferase Assay System* (Promega).

### Structural bioinformatics

Model of the complex between NPM1 and CIGB-300 was created in two steps. Tat part of the peptide was first docked to the structure of NPM1 (PDB ID: 4N8M) [36] with the help of Haddock web portal (http://haddocking.org/). Acidic residues on three subunits were defined as active residues for docking while the setup of the Easy interface was used. Docked pose with best score that enables building of cyclic part of the peptide was then used in the second step. Model of the cyclic peptide was first generated and then placed with program CHARMm [37] in different orientations and positions on the surface of NPM1 in a way that enabled its interaction with the Tat portion of the peptide construct. After linking, the geometry of whole complex was optimized by energy minimization applying 500 steps of steepest descent method. Complex with lowest minimized energy was used as a final mode.

## Results

### HIV-1 Rev directly binds to two distinct regions of NPM1

**Fig 2:**
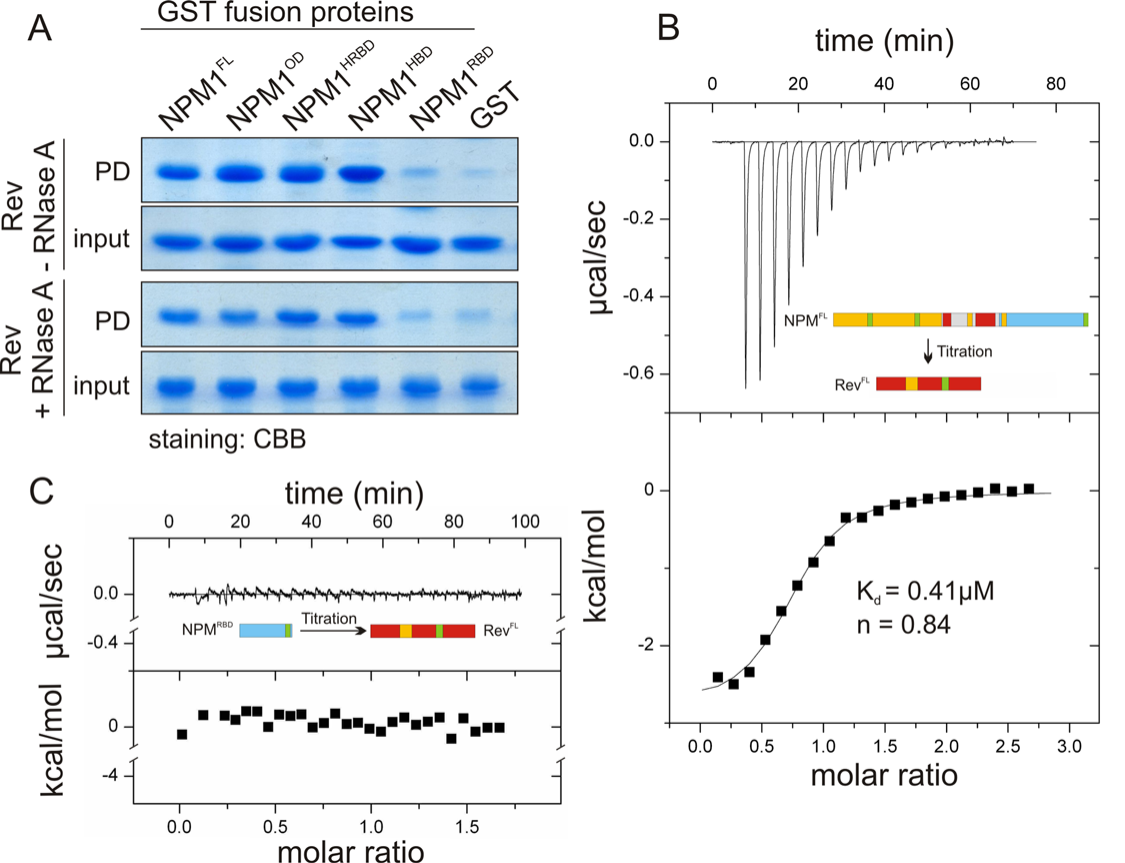
Direct NPM1 interaction with HIV-1 Rev. (A) Qualitative interaction analysis by GST pull-down assay and subsequent CBB staining. NPM1 FL, OD and HRBD, but not RBD, displayed a selective interaction with HIV-1 Rev (upper panel), which was also observed after an RNase A treatment (lower panel). (B) Quantitative interaction analysis by ITC. The binding parameters for the interaction between NPM1^FL^ and Rev were obtained using ITC. Titration of NPM1^FL^ (750 μM) to Rev^FL^ (35 μM) showed an exothermic response (negative peaks) indicating that Rev selectively interacts with NPM1^FL^. The upper graph shows calorimetric changes plotted versus the time and the lower graph represents the changes in temperature according to the molar ratio of the interacting proteins. (C) No interaction was observed in a control experiments by titrating NPM1^RBD^ (300 μM) to Rev^FL^ (30 μM).

Previous reports have shown that NPM1 is co-localized and co-immunoprecipitated with HIV-1 Rev in cells [4, 38]. To investigate a direct interaction between NPM1 and Rev, pull-down experiments under cell-free conditions were performed using Rev^FL^ and NPM1 variants as GST-fusion proteins. As indicated in Fig 2A (upper panel), Rev^FL^ interacts with NPM1^FL^, NPM1^OD^, NPM1^HBD^ and NPM1^HRBD^, but not with the NPM1^RBD^, suggesting that two different regions of NPM1, namely OD and HBD, have tight physical interaction with the HIV-1 Rev. To show whether this interaction is RNA-dependent, the pull-down experiments were performed under the same conditions in the presence of RNase A. As shown in Fig 2A (lower panel), RNase treatment had no effect on HIV-1 Rev association with NPM1. These results clearly indicate that HIV-1 Rev specifically binds to NPM1, and the binding is not RNA-dependent.

Next, we purified all proteins in high quantities (Fig 1B), and after cleaving the tag, isothermal titration calorimetry (ITC) experiments were conducted in order to examine the stoichiometry of binding and to determine the binding affinity of Rev^FL^ for the NPM1 variants. Consistent with the data obtained by pull-down assay, Rev^FL^ revealed variable affinity for the NPM1 variants with calculated dissociation constants (K_d_) between 18 and 0.013 μM for 1:1 stoichiometry (Fig 2B and S1 Fig; Table 2). No interaction was detected between Rev^FL^ and NPM^RBD^ (Fig 2C) suggesting that a low micromolar affinity for the interaction between Rev and NPM1^HRBD^ actually stems from the central histone binding domain of NPM1 (NPM1^HBD^). The obtained dissociation constant (K_d_) for the Rev^FL^ and NPM1^HBD^ interaction was 5.8 μM indicating a stronger affinity for Rev^FL^ as compared to that of NPM^HRBD^, which could be due to a binding site that partially masked by the C-terminal RBD.

**Table 2:** ITC data for HIV-1 Rev^FL^ interaction with NPM1 variants.

Protein	K_d_ (μM)^a^	∆H (kcal/mol)	T∆S (kcal/mol)	n (sites)
NPM1^FL^	0.41	-16.50±0.47	-0.66	0.84
NPM1^OD^	0.013	-3.79±0.11	-0.58	0.94
NPM1^HRBD^	18	-3.23±0.31	-0.27	0.76
NPM1^HBD^	5.8	-1.71±0.20	-0.71	0.85
NPM1^RBD^	no binding	-	-	-

K_a_^,^ association constant; K_d_^,^ dissociation constant; ΔH, enthalpy; n, binding stoichiometry (number of binding sites). HIV-1 Rev^FL^ did not show any binding to the RNA-binding domain (RBD) of NPM1. All measurements were performed at 25°C.

^a^ K_d_ values were calculated from K_d_ = 1/K_a_.

### HSV-1 US11 associates with NPM1 in cells

**Fig 3:**
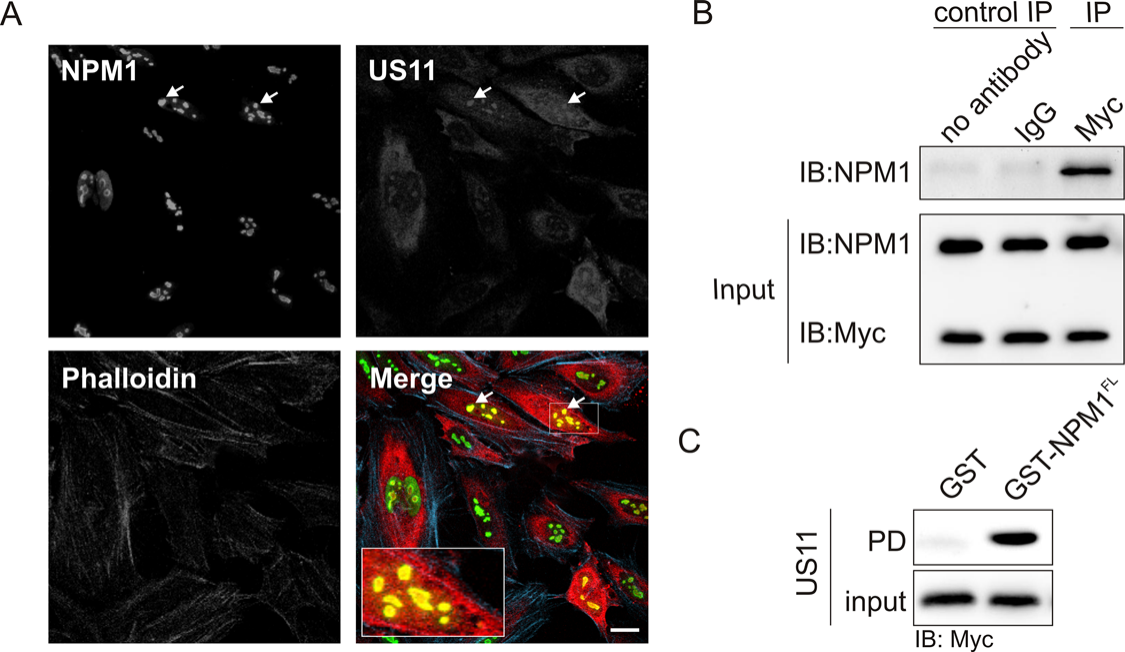
NPM1 association with HSV-1 US11 in the cell. (A) Nucleolar colocalization of endogenous NPM1 with myc-US11. Confocal images of HeLa cells transfected with myc-US11 were obtained by staining endogenous NPM1 (Mouse anti-NPM1 (ab10530)), myc-US11 (anti-myc antibody), and filamentous actin (rhodamine-phalloidin). For clarity, a boxed area in the merged panel shows colocalization of NPM1 and US11 in the nucleolus as pointed by arrows. Scale bar: 20 μm. (B) Myc-US11 associates with endogenous NPM1 in COS-7 cells. NPM1 was co-immunoprecipitated with myc-US11 overexpressed in COS-7 cells using anti-myc antibody. A normal Rabbit IgG and sample without antibody were used as IP controls. Input, 5% of total cell lysate; IP, immunoprecipitation; IB, immunoblotting. (C) Myc-US11^FL^ displayed an interaction with NPM1^FL^. Myc-US11^FL^ was pulled down with the GST-fusion NPM1^FL^, but not with GST, which was used as a negative control. Samples prior pull-down (PD) analysis were used as input control.

The fact that Rev physically binds to NPM1 and US11 alone can fulfill Rex and Rev’s function in transactivating envelope glycoprotein gene expression [14], led us to examine a potential US11-NPM1 interaction. We first analyzed the intracellular distribution of endogenous NPM1 and overexpressed myc-US11 in HeLa cells using confocal imaging. Fig 3A shows a nucleolar co-localization of NPM1 and US11 where the overall pattern of these proteins is different. In contrast to a predominant nucleolar localization of NPM1, US11 was found in the cytoplasm and also accumulated, to certain extent, in the nucleoli. To confirm the association of US11 with NPM1, COS-7 cells overexpressing myc-US11 were lysed and endogenous NPM1 was immunoprecipitated. Fig 3B shows that NPM1 co-precipitated with myc-US11 indicating that US11 forms a complex with NPM1. We, next, used purified GST-NPM1^FL^ and pulled down myc-US11, transiently overexpressed in COS-7 cells. As shown in Fig 3C, the myc-US11^FL^ clearly bound to NPM^FL^, but not to the GST control, indicating that there may be a direct interaction between US11 and NPM1.

### US11 associates with NPM1^OD^ in its oligomeric state

**Fig 4:**
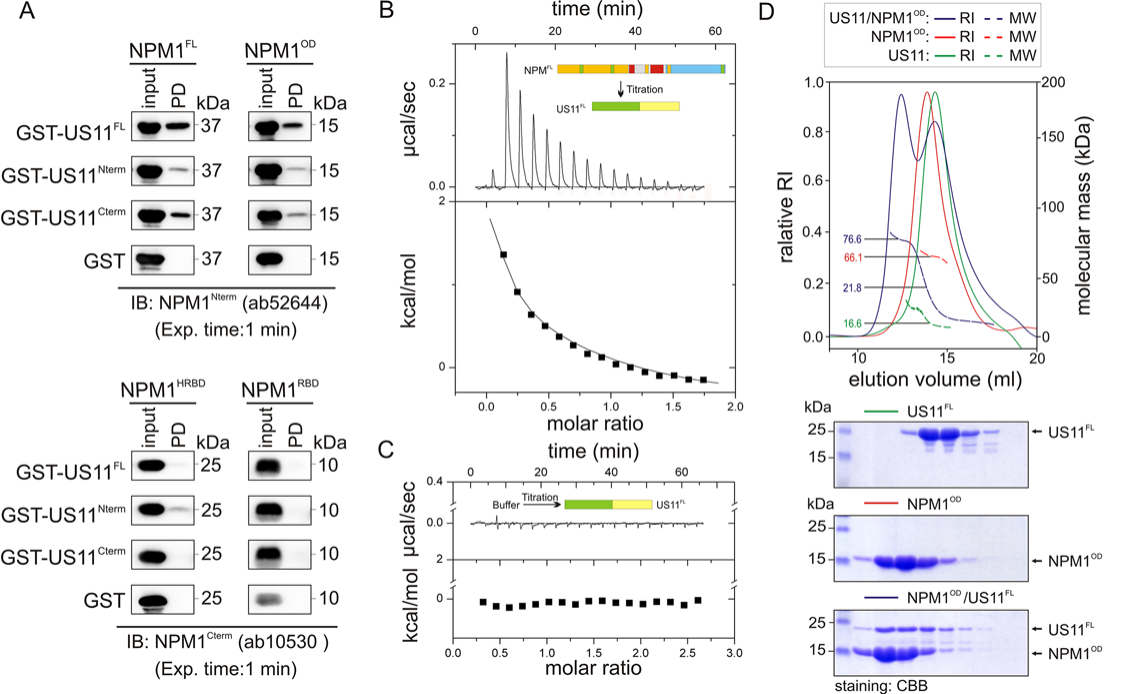
Physical interaction of HSV-1 US11 with NPM1. (A) C-terminal region of US11 largely contributes to NPM1 interaction. Pull-down experiments were conducted with purified proteins in the presence of RNase A by using GST-fused US11^FL^, US11^Nterm^, US11^Cterm^, and GST as a negative control. For the detection of NPM1 variants two different antibodies were used, ab52644 recognized an N-terminal epitope containing in NPM1^FL^ and NPM1^OD^, and ab10530 recognized a C-terminal epitope containing in NPM1^HRBD^ and NPM1^RBD^. The same pattern of interaction was obtained for the N-terminal and the C-terminal parts of US11, although the interaction between NPM1^FL^ and NPM1^OD^ with US11^Nterm^ was much weaker than with US11^Cterm^. The exposure time was 1 min for all the blots. (B-C) US11 binds NPM1 with a binding constant in the low micromolar range. To measure the binding parameter for the NPM1-US11 interaction, 1.2 mM NPM1^FL^ (B) and buffer (C) were titrated to 60 μM US11^FL^. Both NPM1 and US11 were treated with RNase A. Conditions were the same as described in Fig 2. US11 binding to NPM1 is an endothermic reaction. (D) US11 binds to a pentameric NPM1. aSEC-MALS/RI analysis of NPM1^OD^, US11^FL^, and a mixture of both proteins revealed an oligomeric nature of NPM1^OD^ with a molecular weight (MW) of 66.1 kDa corresponding to the pentameric form. Obtained MW for US11 was 16.6 kDa, which matches the theoretical MW of 16.7 kDa for a monomeric US11 (upper panel). SDS-PAGE and CBB staining of the aSEC (Superdex 200, 10/300) elution fractions of NPM1^OD^, US11^Fl^, and a mixture of both clearly revealed a NPM1-US11 complex formation (lower panel). Both NPM1 and US11 were treated with RNase A. The MW of this complex corresponds to 76.6 kDa for a pentameric NPM1^OD^, and a monomeric US11^FL^. A MW of 21.8 kDa was measured that is estimated to an unbound US11^FL^.

To clarify whether the interaction observed above is a direct interaction, we used purified, RNase A treated NPM1 and US11 variants from *E*. *coli*. Fig 4A shows that NPM1^FL^ and NPM1^OD^ but not NPM1^HRBD^ and NPM1^RBD^, directly interact with US11^FL^. We repeated the experiments to map the NPM1 binding region of US11 by using purified, GST-fused, N-terminal and C-terminal fragments of US11. As shown in Fig 4A, both US11^Cterm^ and US11^Nterm^ bound, with the same pattern as US11^FL^ bound to NPM1^FL^ and NPM1^OD^. However, binding affinities of isolated N- or C-terminal domains of US11 towards NPM1 seemed markedly reduced compared to the full-length protein. In the light of above mentioned, we conclude that NPM1 and US11 physically interact with each other *via* NPM1^OD^ and largely US11^Cterm^.

Next, ITC measurement was also performed to determine the binding affinity between NPM1 and US11 by titrating NPM1^FL^ (1.2 mM) to US11^FL^ solution (60 μM); both proteins were treated with RNase A. As shown in Fig 4B, the association of NPM1^FL^ with US11^FL^ is endothermic (positive peaks). As a control experiment, buffer was titrated to 60 μM US11^FL^ under the same experimental condition with no calorimetric changes (Fig 4C). Based on ITC analysis we estimated an apparent K_d_ value of 4 μM. The NPM1^OD^ interaction with US11^FL^ was also analyzed by aSEC combined with MALS, after treating the proteins with RNase A. Fig 4D (lower panel) shows a co-elution of the RNase-treated NPM1^OD^ and US11^FL^ proteins from the Superdex 200 (10/300) column indicating that these proteins form a complex. MALS analysis revealed that NPM1^OD^ oligomerized to a pentameric state and formed a 1:1 complex with the monomeric US11^FL^ (Fig 4D upper panel). To further investigate the oligomerization states of US11 and NPM1, AUC experiments were performed. Results obtained were consistent with the MALS data, and revealed that NPM1^FL^ and NPM1^OD^ are pentameric and globular while US11^FL^ was monomeric and adopts an elongated structure (Table 3 and S2 Fig). Together, the data clearly demonstrates that US11 selectively binds to the N-terminal oligomerization domain of NPM1 in an RNA-independent manner.

**Table 3:** AUC-SV data for NPM1^FL^, NPM1^OD^, and US11^FL^, respectively.

Proteins	S_20,w_ (S)	Std. dev.	f/f_0_	MW (kDa)
NPM1^FL^	6.7	0.52	1.5	146
NPM1^OD^	4.5	0.14	1.27–1.40	63.3
US11^FL^	1.4	0.20	1.4–1.7	15.3

MW, molecular weight; S20,w (S), sedimentation rate at 20°C; f/f0, frictional coefficient. In all three cases the values refer to a single, dominant species, which represented more than 90% of the sample.

### Displacement of the NPM1-CIGB-300 complex by Rev and US11

**Fig 5:**
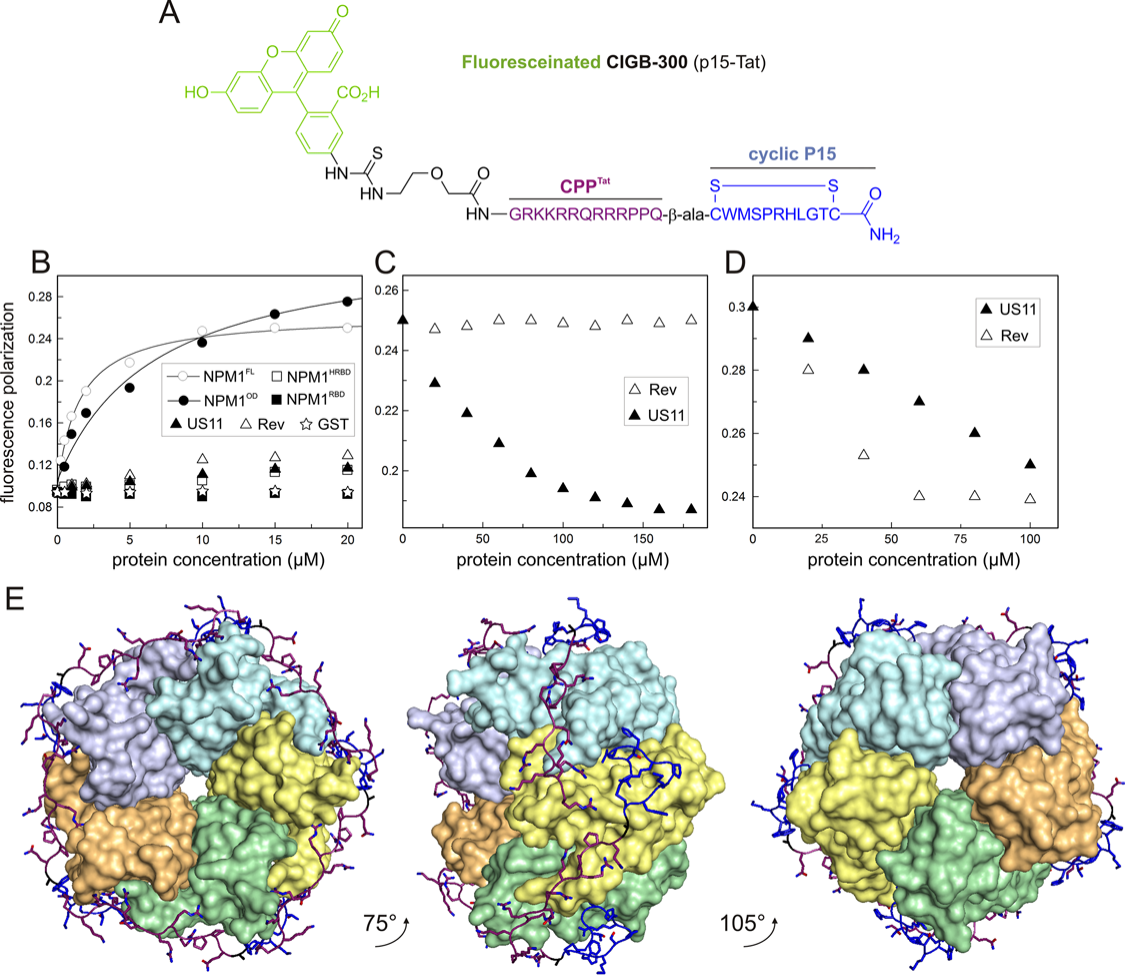
The synthetic peptide CIGB-300 competes with Rev and US11 by binding NPM1^OD^ with high-affinity. (A) CIGB-300 consists of the cyclic P15 (blue) and the Tat (purple) peptides, and labeled with fluorescein (green; FITC). (B) Fluorescence polarization experiments conducted by titrating increasing amounts of NMP1 variants, Rev, US11, and GST to 0.1 μM FITC-labelled CIGB-300 (f CIGB-300). A high affinity interaction with the peptide was only observed for NPM1^FL^ and NPM1^OD^, resulting from an increase of polarization, but not for Rev, US11, GST, and the other NPM1 variants. (C-D) Contrary to US11, Rev only displaced NPM1^OD^ from its fCIGB-300 complex. Displacement experiments were performed by adding increasing amounts of Rev or US11 to the NPM1^FL^-fCIGB-300 complex (C) or to the NPM1^OD^-fCIGB-300 complex (D). (E) A proposed NPM1^OD^-CIGB-300 docking model of pentameric NPM1^OD^ structure in the complex with CIGB-300. Cyclic part (blue) and basic part (purple) of the peptide shown as sticks and ribbons wraps around several monomeric units of NPM1 represented by surfaces in different colors shown in top view (left), rotated orientation (middle), and the bottom view (right).

Synthetic peptide CIGB-300 (also called p15-Tat; Fig 5A) has been described as a proapoptotic and anti-cancer peptide, which directly targets and antagonizes NPM1 function in cancer cells [39, 40]. Fluorescence polarization analysis revealed that a FITC-labelled CIGB-300 tightly associates with NPM1^FL^ and NPM1^OD^ but not with NPM1^HRBD^ and NPM1^RBD^ (Fig 5B). Calculated K_d_ values for the FITC-labelled CIGB-300 interaction with NPM1^FL^ and NPM1^OD^ were 1.4 and 6.6 μM, respectively.

We used the NPM1^FL^- FITC-labelled CIGB-300 complex to further investigate NPM1 interactions with Rev and US11. The idea here was that titrating Rev or US11 to the complex may result in displacement of NPM1^FL^ from the FITC-labelled CIGB-300. Fig 5C shows that increasing concentrations of US11, but not Rev, significantly displaced NPM1^FL^ from the FITC-labelled CIGB-300 complex. This result was surprising for two reasons: First, Rev binds NPM1 in a higher nanomolar range (Table 2) and should be able to compete with CIGB-300 provided that both bind to the same surface of the NPM1 protein. Interestingly, Rev revealed a 30-fold lower affinity for NPM1^FL^ as compared to NPM1^OD^ (Table 2), which may explain why Rev did not displace NPM1^FL^ from FITC-labelled CIGB-300. Second, US11, which evidently exhibits an approximately 10-fold lower binding affinity for NPM1^FL^ as compared to Rev, is able to displace NPM1^FL^ from its complex with the synthetic FITC-labelled CIGB-300 (Fig 5C). To address this issue we repeated the displacement experiments under the same conditions as before but used the FITC-labelled CIGB-300 complex with NPM1^OD^ instead of NPM1^FL^. Data obtained revealed that both Rev and US11 efficiently displace FITC-labelled CIGB-300 by binding to NPM1^OD^ (Fig 5D), indicating that Rev, US11 and FITC-labelled CIGB-300 have overlapping binding sites on NPM1^OD^.

To obtain a first structural assessment of NPM1^OD^ site targeted by CIGB-300 we conducted a multistage protein-ligand docking approach. Assuming that basic part of CIGB-300 determines the binding, its Tat tail was docked in the first step. In the second step, the cyclic part was placed on the surface of NPM1^OD^ and linked to the peptide fulfilling geometry and energy criteria. Whole peptide contacted three out of five monomeric units of the pentameric NPM1^OD^, but in a way that enables five copies of CIGB-300 to be generated without sterical clashes (Fig 5E). It is important to note that a stoichiometry of 1:1 emerged spontaneously, as the criteria that five peptides should bind to NPM1^OD^ pentamer was not applied while generating of the model. The feature that CIGB-300 wraps around at least several monomeric units (Fig 5E, middle panel) points to a stabilization effect of bound peptides and is consistent with the model of NPM1 in complex with R-rich proteins, such as p19^ARF^, ARF6, Rev and the ribosomal protein L5 [36].

### HIV-1 production is influenced in CIGB-300 treated cells

**Fig 6:**
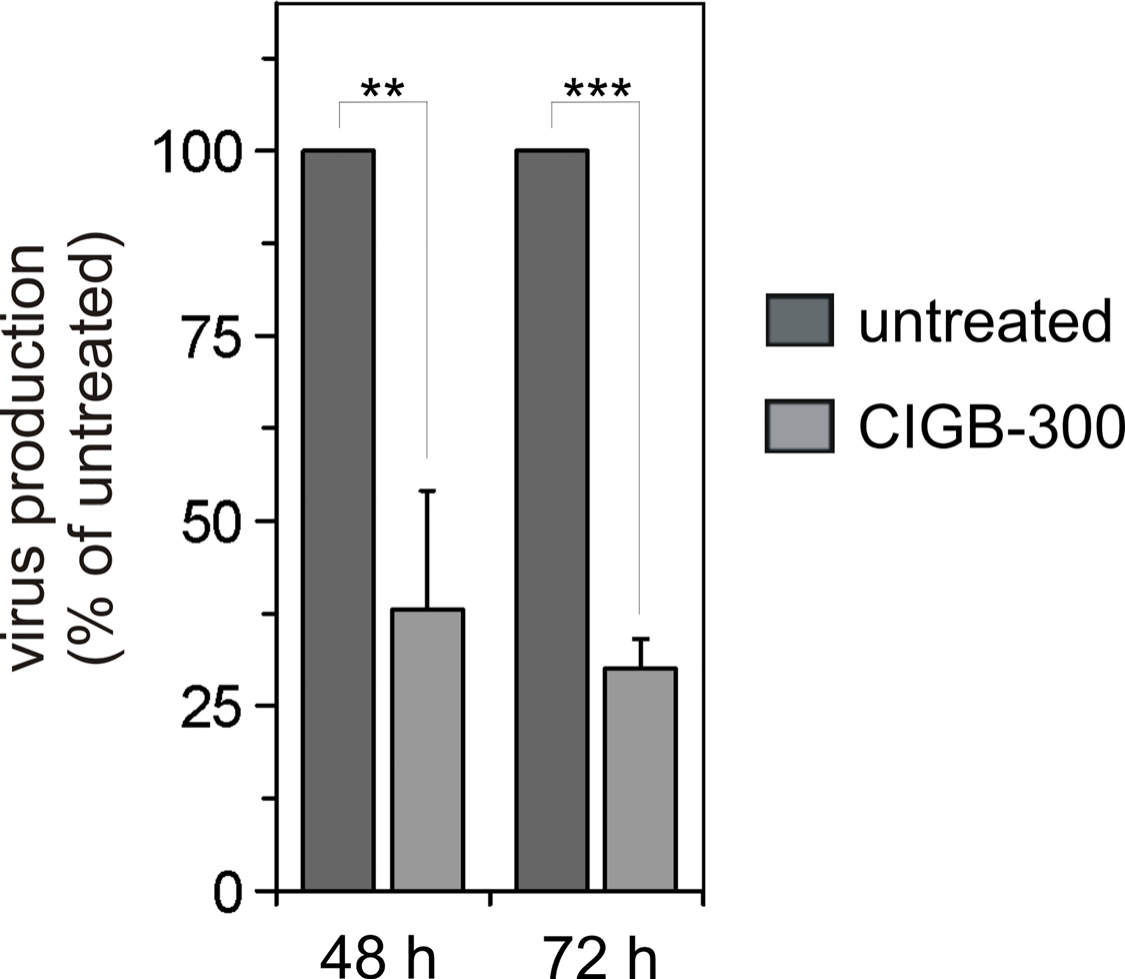
CIGB300 treatment interferes with HIV-1 production. CIGB-300 treated or untreated HOS.CD4.CXCR4 cells were infected with NL4.3 virus at an MOI of 1. Culture supernatant was collected 48 and 72 h post infection and virus titer was determined. The figure shows one representative experiment out of four, in which virus quantification was performed by TZM-bl cell titration. Values are the means ± S.D. of three measurements. Statistical significance (P) was calculated by the Student`s t-test: ***P<0.002; **P<0.02.

In order to investigate the possible role of NPM1-Rev interaction for HIV-1 replication, HOS-CD4.CXCR4 cells were incubated with CIGB-300 for 30 min or left untreated. After removing the peptide, cells were infected with HIV-1 (clone NL4.3, MOI 1). Culture supernatants were collected 48 and 72 h post infection and were quantified by titration on the HIV-1 reporter cells TZM-bl. In cells treated with CIGB-300, the virus production was reduced by 63% and 70% after 48 h and 72 h post infection, respectively (Fig 6). Thus, CIGB-300 may interfere with an NPM1-Rev interaction in cells and affect Rev-dependent gene expression and subsequently HIV infection.

## Discussion

Since its discovery 34 years ago, intensive research has been performed on NPM1. NPM1 is ubiquitously expressed and significantly upregulated in response to cellular stress signals [18, 19, 41, 42] leading to the alteration of nucleolar structures and its re-localization to other cellular compartments. As a global effector, it has been implicated in maintenance of genomic stability, transcriptional gene regulation, ribosome biogenesis, centrosome duplication, DNA repair, control of cellular senescence, protection against radiation-induced apoptosis, tumor suppression, and has been increasingly emerging as a potential cellular factor for viral infection (see Table 1). Most of these functions have hitherto remained obscure and unexplained.

To shed light on the association of NPM1 with viral proteins, we have investigated its physical interaction with HIV-1 protein Rev and HSV-1 protein US11. Based on our results Rev exhibits affinity towards two NPM1 binding sites: on the pentameric, N-terminal oligomerization domain (NPM1^OD^) and on the central histone-binding domain (NPM1^HBD^), while HSV-US11 has only one binding site on NPM1^OD^. We suggest that the different NPM1 domains interact in a mechanistically different mode with the Rev and US11 proteins. Rev association with NPM1 is the result of presumably an RNA-independent bimodal binding mechanism, according to our data, of (i) a low-affinity binding to the histone-binding domain of NPM1 (K_d_ = 5.8 μM) and (ii) a very high-affinity binding to oligomerization domain of NPM1 (K_d_ = 0.013 μM), leading to an overall K_d_ value of 0.4 μM for the full-length NPM1 (Table 2). In the case of the NPM1-US11 interaction, we observed a strong binding of US11 to NPM1^OD^, which is most probably achieved *via* its C-terminal RBD (US11^Cterm^; See Figs 1A and 4A). While the data regarding US11 reports its unprecedented direct interaction with NPM1, our measurements with Rev confirm previously obtained observations. It has been shown that two different transcripts of NPM1, B23.1 and B23.2, prevent the aggregation of Rev *via* their proposed chaperone activity [43]. B23.1, which was also used in this study, is identical to B23.2 but has a 35-amino acid longer C-terminus. As the prevention of Rev aggregation by both constructs was nearly identical, this C-terminus was excluded from the interaction with Rev [43], which is in agreement with our results from PD and ITC experiments (Fig 2 and S1 Fig; Table 2). Our finding of a 1:1 ratio (n≈ 0.84) between NPM1 and Rev obtained by ITC (Table 2) is also consistent with earlier studies that have suggested a stoichiometric interaction between NPM1 and Rev, and a maximal stimulation of the import of Rev into the nucleus by NPM1 at a 1:1 molar ratio [4, 43]. This stoichiometric ratio suggests that NPM1^FL^ exhibits one binding site for one HIV-1 Rev molecule. Since Rev has the tendency to aggregate also under normal physiological conditions [44], it is very likely that NPM1, by acting as a molecular chaperone, increases Rev’s solubility and mobility during the import into and throughout the nucleus.

US11 is an abundant HSV-1 protein, which is expressed late during infection [45]. It has been reported that US11 functionally substitutes Rev and Rex proteins by stimulating expression of glycoproteins required for retroviral envelope synthesis [14]. US11 interaction with cellular proteins may, therefore, be required during HSV-1 infection. However, so far, only a few proteins including 2'-5'-oligoadenylate synthetase [46], cellular kinesin light-chain-related protein PAT1 [45], human ubiquitous kinesin heavy chain [24], protein kinase R (PKR) [47], protein activator of the interferon-induced protein kinase (PACT) [48], and nucleolin [23] have been reported. NPM1 and nucleolin are among the most abundant nucleolar proteins [5] with high functional but not structural similarities. They are usually found in the granular components and dense fibrillar components of nucleoli, have the same distribution as US11 [49], and are re-localized during HSV-1 infection [7, 50]. With NPM1, we have identified in this study a new nucleolar protein partner for US11 and characterized the subdomains responsible for their interactions. US11 has two domains (Fig 1A): An N-terminal domain called effector domain (ED) and a C-terminal RNA-binding domain (RBD). C-terminal domain consisting of 20–24 XPR (X, any amino acid; P, proline; R, arginine) repeats has a polyproline type II helix organization and is usually engaged in interactions with other proteins [15]. US11^ED^ is necessary for transactivation of gene expression, transport, and mRNA translation [15]. Therefore, we designed two deletion variants of US11 (N- and C- terminus) to determine the part involved in the interaction with NPM1. In contrast to nucleolin, which has been reported to interact with the C-terminus of US11 [23], our data clearly shows that both domains are apparently required for the interaction with NPM1. The C-terminal domain of US11, which is involved in the nucleolar localization of US11, binds to NPM1 stronger than the N-terminal domain (Fig 4A). Since C-terminus of US11 is rich in arginine, these results support the idea that arginine-rich motif (R-rich) mediates the interactions with NPM1 [36]. Synthetic peptide CIGB-300 used in our investigation also falls into this category as it is the conjugate of R-rich peptide Tat, and the cyclic peptide (hence is called p15-Tat; Fig 5A). This peptide, which has been described as a proapoptotic peptide with antiproliferative activity *in vitro* and antitumoral activity *in vivo* [51], has been reported to directly bind to NPM1 [39, 40]. We observed in this study that only NPM1^OD^, but not the other domains of NPM1, associates with fCIGB-300. Interestingly, the K_d_ value for the fCIGB-300 interaction with NPM1^FL^, derived from our polarization measurements (Fig 5B), was indicative of almost 5-fold higher affinity than that of fCIGB-300-NPM1^OD^ interaction. This higher affinity can be explained by an avidity effect that originates from core N-terminal domain and the dynamic flexible tails, similarly to the model proposed for nucleoplasmin interaction with histones [52]. NPM1^OD^ is followed by the two highly acidic regions with disordered structure and a C-terminal RBD that folds as a three-helix bundle [53]. The biological significance of the acidic regions (A1-A3; Fig 1A) has not been established. The A1 region in NPM1^OD^ has been recently shown to play a crucial role in the interaction with R-rich motifs of NPM1 binding proteins, such as p19ARF, ARF6, the ribosomal protein L5, and HIV1 Rev [36]. A model of the complex between NPM1^OD^ and CIGB-300 provided insights into different sites for the association of the CIGB-300 peptide, especially the R-rich motif of the CPP^Tat^ contacting negative charges of the A1 region of NPM1^OD^ (Figs 1A and 5E). Additionally, our displacement experiment with Rev indicates that CIGB-300 shares the same binding site on NPM1 and may act as an inhibitor of NPM1-Rev interaction. Most likely for the same reason, we observed a reduced expression of viral production in HIV-1 infected cells treated with the CIGB-300 peptide (Fig 6).

Furthermore, our displacement data shows that the NPM1-US11 interaction was also modulated by CIGB-300 (Fig 5C and 5D). Thus, it is tempting to speculate that US11 and Rev, two functionally homologous viral proteins, share a similar binding site on NPM1 as suggested in this study for CIGB-300. An amino acid sequence analysis revealed clear differences in the R-rich motifs between Rev (^38^RRNRRRRWRARAR^48^) and US11, which consists of 21 `XPR´ repeat motifs in US11^Cterm^. R-rich motifs act as NLS by binding to the nuclear import receptors in nuclear translocation of viral proteins [10, 12, 54, 55]. On the other hand, nucleolar shuttling and accumulation of Rev requires interaction with NPM1 [4, 12]. US11 is similarly shuttling between the nucleus and the cytoplasm in transiently transfected cells and HSV-1-infected cells [20, 56]. Mutagenesis and modeling studies of the C-terminus of US11, containing XPR repeats, have shown that this region is critical for both nucleolar accumulation of US11 and its nucleocytoplasmic export [15, 57]. As mentioned above, CIGB-300 has the cell penetrating peptide Tat with R-rich motif, which corresponds to the presumed nuclear localization signal (NLS). Tat moves across the nuclear envelope and consequently drives CIGB-300 to the nucleus. Thus, we hypothesize that, (i) R-rich motifs of viral proteins serve as NPM1 binding sites that facilitate their nuclear transport analogous to NLS-importin system, and (ii) NPM1 most likely acts as an auxiliary factor for R-rich motif-containing viral proteins, such as HIV-1 Rev and HSV-1 US11, and achieves their transport into different nuclear compartments and subnuclear domains, leading to nuclear egress of infectious viral particles. Thus, NPM1 seems to represent a key protein in viral infections that is hijacked by invading pathogens to facilitate infection. As a consequence, NPM1 may represent a novel promising target for antiviral therapeutic intervention.

## Supporting Information

S1 Fig Physical interaction of HIV-1 Rev with NPM1. Quantitative interaction analysis were performed by ITC at 25°C by titrating (A) NPM1OD (450 μM) to 30 μM HIV-1 Rev, (B) NPM1HBD (350 μM) to 25 μM HIV-1 Rev and (C) NPM1HRBD (800 μM) to 50 μM HIV-1 Rev, respectively. The upper graph shows calorimetric changes plotted versus the time, and the lower graph represents the changes in temperature according to the molar ratio of the interacting proteins.
(TIF)

S2 Fig Analytical ultracentrifugation for the determination of the oligomeric state and molecular mass of US11 and NPM1. (A) Sedimentation velocity analysis of US11^FL^ and NPM1^FL^ at 35,000 rpm and 20°C. Graphs show the evaluated c(s) distributions obtained by SEDFIT. For presentation, curves had been normalized to maximum peak height. Results revealed that NPM1^FL^ and US11^FL^ are pentameric and monomeric, respectively. (B) The left panel contains data obtained from the sedimentation velocity analysis of NPM1^OD^, which shows the population of pentamer, and the right panel are data obtained from sedimentation equilibrium analysis of 0.25 μM NPM1^OD^ at 14000 (purple), 16000 (blue), 25000 (cyan), 42000 (green) and 50000 rpm (yellow) at 20°C. Experimentally determined concentration profiles were fitted globally with a single species model resulting in a molecular mass of 65180 ±640 Da corresponding to a pentamer of NPM1^OD^. The experimental data together with the fitted concentration profiles are shown on the top, and at the bottom, residuals from the fit are documented.
(TIF)

## Abbreviations

aa: amino acid
A1-A3: acidic regions 1–3
aSEC: analytical SEC
CBB: Coomassie Brilliant Blue
Cterm: C-terminal
f: fluoresceinated
FL: full-length
HSV-1: herpes simplex virus type 1
HIV-1: human immunodeficiency virus type 1
HRBD: histone and RNA-binding domains
IP: immunoprecipitation
ITC: isothermal titration calorimetry
MALS: multi angle light scattering
NES: nuclear export signal
NLS: nuclear localization signal
NoLS: nucleolar localization signal
NPM1: nucleophosmin
Nterm: N-terminal
OD: oligomerization domain
PD: pull-down
RBD: RNA-binding domain
SEC: size exclusion chromatography
