# Supplementary figures and images for: Biophysical Characterization of Nucleophosmin Interactions with Human Immunodeficiency Virus Rev and Herpes Simplex Virus US11
Source: PLoS One. 2015 Dec 1;10(12):e0143634. doi: 10.1371/journal.pone.0143634 (PMC4704560; doi:10.1371/journal.pone.0143634)

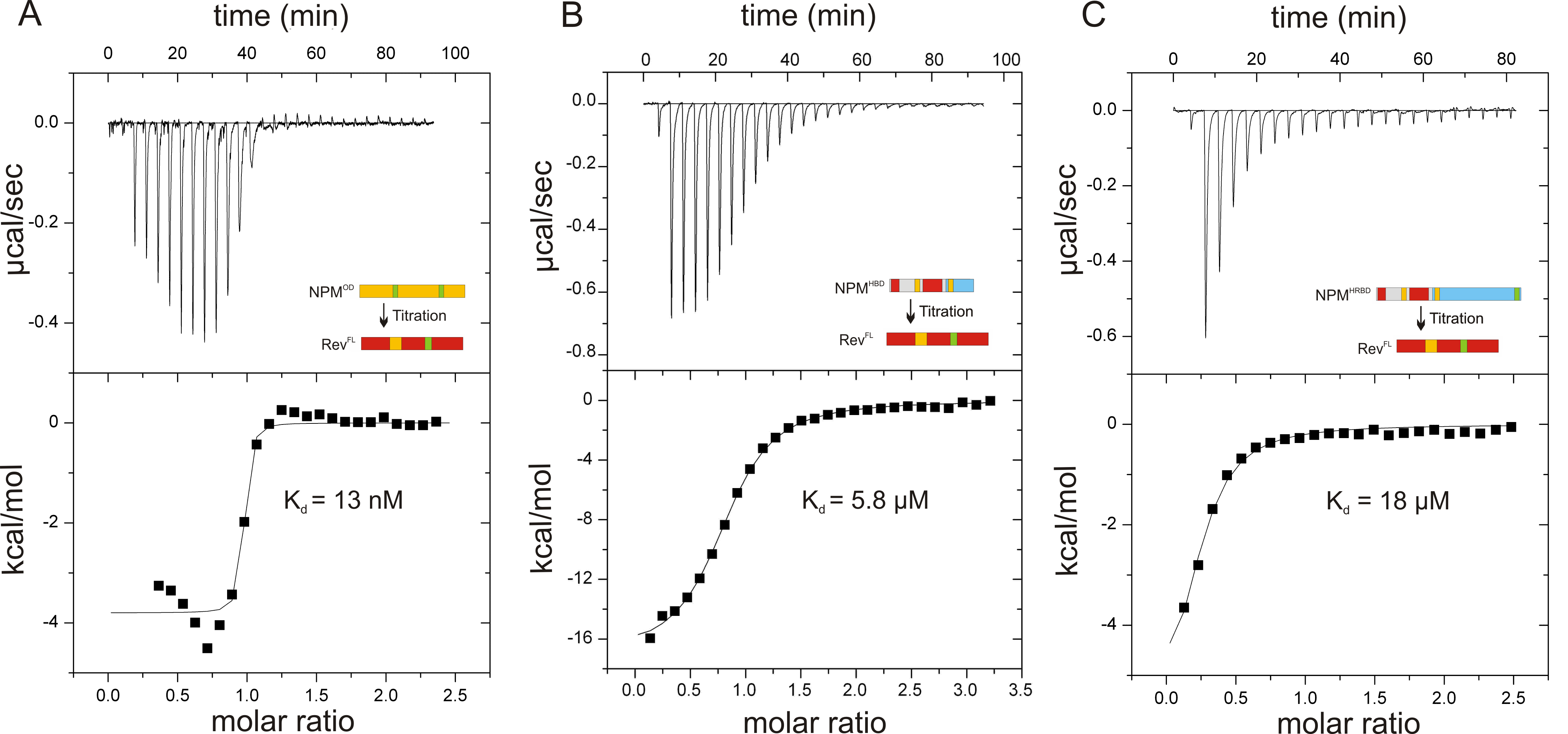

Supplement: S1 Fig — Quantitative interaction analysis were performed by ITC at 25°C by titrating (A) NPM1OD (450 μM) to 30 μM HIV-1 Rev, (B) NPM1HBD (350 μM) to 25 μM HIV-1 Rev and (C) NPM1HRBD (800 μM) to 50 μM HIV-1 Rev, respectively. The upper graph shows calorimetric changes plotted versus the time, and the lower graph represents the changes in temperature according to the molar ratio of the interacting proteins. (TIF) [file pone.0143634.s001.tif]

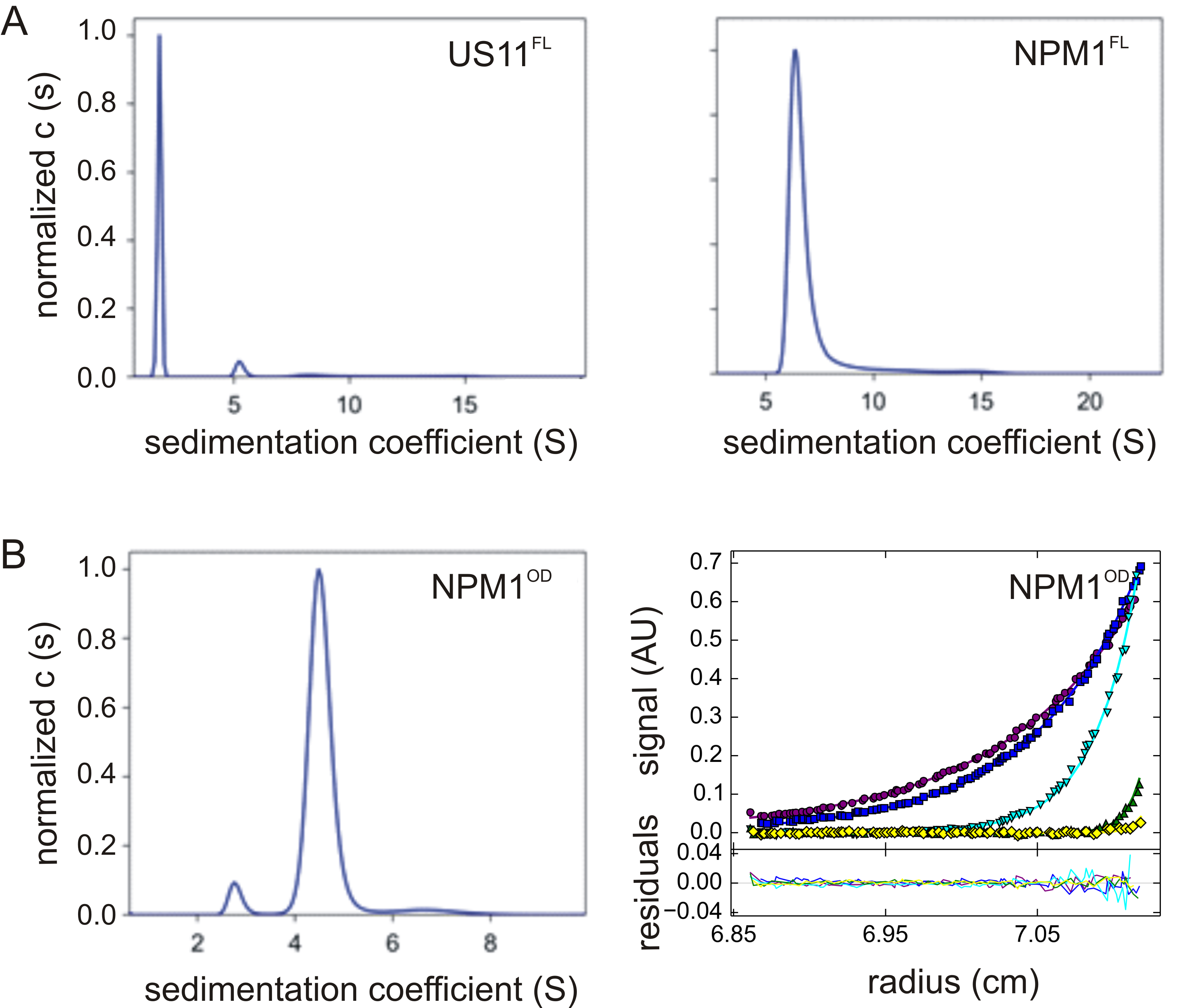

Supplement: S2 Fig — (A) Sedimentation velocity analysis of US11FL and NPM1FL at 35,000 rpm and 20°C. Graphs show the evaluated c(s) distributions obtained by SEDFIT. For presentation, curves had been normalized to maximum peak height. Results revealed that NPM1FL and US11FL are pentameric and monomeric, respectively. (B) The left panel contains data obtained from the sedimentation velocity analysis of NPM1OD, which shows the population of pentamer, and the right panel are data obtained from sedimentation equilibrium analysis of 0.25 μM NPM1OD at 14000 (purple), 16000 (blue), 25000 (cyan), 42000 (green) and 50000 rpm (yellow) at 20°C. Experimentally determined concentration profiles were fitted globally with a single species model resulting in a molecular mass of 65180 ±640 Da corresponding to a pentamer of NPM1OD. The experimental data together with the fitted concentration profiles are shown on the top, and at the bottom, residuals from the fit are documented. (TIF) [file pone.0143634.s002.tif]
